# MUC1 peptide-loaded dendritic cell vaccine boosts antitumor immunity in pancreatic cancer

**DOI:** 10.3389/fimmu.2025.1752861

**Published:** 2026-01-13

**Authors:** Huiping Xie, Wenzhuo Yang, Haodong Chen, Zhilan Zhang, Zelin Zhao, Yuanyuan Jin, Shuai Fan, Zhaoyong Yang

**Affiliations:** 1 The School of Basic Medical Sciences, North China University of Science and Technology, Tangshan, China; 2 The Department of Oncology, Beijing Hospital, National Center of Gerontology, Institute of Geriatric Medicine, Chinese Academy of Medical Sciences, Beijing, China; 3 National Health Commission (NHC) Key Laboratory of Biotechnology of Antibiotics, Institute of Medicinal Biotechnology, Chinese Academy of Medical Sciences, Beijing, China; 4 The School of Pharmacy, North China University of Science and Technology, Tangshan, China

**Keywords:** dendritic cell vaccines, humanized mice, MUC1, pancreatic cancer, umbilical cord blood

## Abstract

**Objective:**

Pancreatic cancer is one of the most aggressive malignancies with a poor prognosis and limited treatment options. This study aimed to evaluate the efficacy of a dendritic cell (DC) vaccine pulsed with mucin 1 (MUC1) peptide antigens in the immunotherapy of pancreatic cancer.

**Methods:**

Mononuclear cells were isolated from umbilical cord blood and induced to differentiate into DCs. The surface markers of DCs and their phagocytic capacity for FITC-OVA were detected using flow cytometry. The stimulatory effect of DC vaccines loaded with MUC1 antigen peptides (568 and 619) on T lymphocyte proliferation was assessed by CCK-8 assay. ELISA was used to measure the secretion of IL-12p70 by DCs and IFN-γ production by activated cytotoxic T lymphocytes (CTLs). The proportion of CD8^+^ and CD4^+^ cells among CTLs activated by the DC vaccine was analyzed via flow cytometry. The cytotoxic activity of activated T cells against pancreatic cancer cell lines was evaluated using an LDH release assay. Furthermore, bioinformatic analysis was performed to compare MUC1 expression between pancreatic cancer and normal tissues and its correlation with patient prognosis. Western blot was used to detect MUC1 expression in pancreatic cancer cell lines. The antitumor effect of the DC vaccine pulsed with antigen peptide 619 was investigated in a humanized huHSC-M-NSG mouse model of pancreatic cancer.

**Results:**

Immature DCs (imDCs) highly expressed CD11c, HLA-DR, and CD86, but weakly expressed CD14; mature DCs (mDCs) highly expressed CD11c, HLA-DR, CD83, CD80, and CD86, and weakly expressed CD14. DCs on day 5 of culture exhibited the strongest phagocytic capacity for FITC-OVA. DC vaccines loaded with either MUC1 peptide 568 or 619 significantly promoted T lymphocyte proliferation and induced higher levels of IL-12p70 and IFN-γ secretion. The peptide-pulsed DC vaccines significantly increased the proportion of CD8^+^ T cells among CTLs and mediated dose-dependent cytotoxic effects against pancreatic cancer cell lines (PANC-1, BXPC-3, MIA PaCa-2), with the highest efficacy observed in the MUC1 peptide 619 group. Bioinformatic analysis revealed that MUC1 was highly expressed in pancreatic cancer tissues and associated with poor patient prognosis. Western blot further confirmed MUC1 expression in pancreatic cancer cell lines. *In vivo*, the DC vaccine pulsed with peptide 619 significantly suppressed tumor growth (tumor weight inhibition rate: 51.4%), increased the percentage of CD8^+^ T cells in peripheral blood, and enhanced the infiltration of hCD45^+^ cells into tumor tissues.

**Conclusion:**

MUC1 peptide-pulsed DCs effectively activate specific CTL responses, indicating that DC-based vaccine immunotherapy holds promise for the management of pancreatic cancer.

## 1 Introduction

Pancreatic cancer, one of the most lethal malignancies, has a five-year survival rate of less than 10% and ranks as the fourth leading cause of cancer-related mortality worldwide (1, 2). This disease is characterized by its aggressive nature and frequent late-stage diagnosis, often limiting therapeutic options (3, 4). Current strategies, including surgery, chemotherapy, and radiotherapy, exhibit limited efficacy due to the tumor’s immunosuppressive microenvironment and immune evasion mechanisms (5). Moreover, conventional chemotherapeutic agents are associated with severe side effects, complicating patient management. Despite advancements in oncology, there remains an urgent need for innovative and effective therapies. Immunotherapy, while promising, faces challenges in pancreatic cancer due to the lack of robust immunotherapeutic strategies. DC-based vaccines, which target tumor-specific antigens, represent a novel approach to enhance antitumor immunity (6). However, clinical translation of DC vaccines for pancreatic cancer remains hindered by multiple challenges, necessitating further exploration to realize their potential in improving patient outcomes. This underscores the importance of optimizing immunotherapeutic strategies, particularly DC vaccine-based approaches, to address this devastating disease.

MUC1, a transmembrane glycoprotein, is minimally expressed and heavily glycosylated in normal tissues but aberrantly overexpressed and hypoglycosylated in epithelial tumors such as pancreatic cancer, exposing tumor-specific epitopes and making it an ideal target for immunotherapy (7, 8). The variable number tandem repeat (VNTR) domain of MUC1 consists of a 20-amino acid sequence (PAPGSTAPPAHGVTSAPDTR), repeated 20 to 125 times. Notably, the GVTSAPDTRPAPGSTAP motif within this sequence has been identified as a critical protective epitope for tumor cell recognition, capable of eliciting potent immune responses (9). Preclinical studies further support the ability of MUC1 short peptides to activate T cells and induce antitumor immunity (10, 11). Compared to full-length MUC1 antigenic peptides, short MUC1-derived peptides containing key epitopes enhance DC processing and presentation efficiency, enabling focused activation of specific T-cells. This approach improves immune specificity while minimizing off-target immunosuppression. These findings justify the design of DC vaccines loading MUC1-derived short peptides to overcome immune tolerance and amplify antitumor responses.

This study aimed to construct a novel MUC1-DC vaccine to address the challenges of inconsistent cell sources in traditional DC vaccines and the limited predictive value of preclinical models. To this end, we innovatively employed umbilical cord blood-derived monocytes for DC induction and utilized the huHSC-M-NSG humanized mouse model for systematic evaluation. Monocytes were isolated from human umbilical cord blood and induced into mature DCs, which were then pulsed with MUC1-derived peptides: MUC1 (157-172) (Peptide 568: GVTSAPDTRPAPGSTA) or MUC1 (144-173) (Peptide 619: TRPAPGSTAPPAHGVTSAPDTRPAPGSTAP). Both peptides are MHC-I-restricted epitopes (12–14). The vaccine-induced immune response was comprehensively evaluated through T cell activation assays, *in vitro* anti-tumor activity experiments, and *in vivo* studies in humanized pancreatic cancer mouse models. Furthermore, bioinformatics and molecular biology techniques were integrated to validate the expression pattern of MUC1 in pancreatic cancer and its clinical relevance, thereby providing a theoretical foundation for the targeted design of the DC vaccine. This study seeks to establish a novel immunotherapeutic strategy for pancreatic cancer and to elucidate the mechanisms underlying the intervention of DC vaccines loaded with MUC1 peptides.

## 2 Materials and methods

### 2.1 Mice and cell lines

huHSC-M-NSG mice were purchased from Shanghai Model Organisms Center, Inc. All animal experiments were conducted in accordance with the ethical guidelines approved by the Animal Ethics Committee of the Chinese Academy of Medical Sciences and Peking Union Medical College Hospital (Ethics Approval No. 00001865). Human pancreatic cancer cell lines PANC-1, BXPC-3, and MIA PaCa-2 were obtained from Procell Life Science & Technology Co., Ltd. (Wuhan, China) and cultured in high-glucose DMEM (Gibco), RPMI1640 (Gibco), and DMEM (Gibco) media, respectively, supplemented with 10% fetal bovine serum (FBS, Gibco), 100 U/mL penicillin and 100 μg/mL streptomycin (Gibco). Cells were grown as adherent cultures at 37°C in 5% CO_2_ and passaged after detachment by 0.05% trypsin.

### 2.2 imDCs preparation and antigen uptake assay

Umbilical cord blood samples were collected from healthy full-term deliveries with approval from the Ethics Committee of Zibo Changguo Hospital (Approval No. CGYJ-BD-080-V2). Mononuclear cells were isolated using Ficoll density gradient centrifugation. Cells were resuspended in X-VIVO 15 medium (Lonza) and seeded at 5 × 10^6^ cells/mL in 25 cm² vented culture flasks. After 2 hours of adherence at 37°C with 5% CO_2_, adherent cells (DC precursors) and non-adherent cells (mixed lymphocytes) were separated. Adherent cells were then cultured in X-VIVO 15 medium containing granulocyte–macrophage colony-stimulating factor (GM-CSF) (50 ng/mL, North China Pharmaceutical Group Co., Ltd.), interleukin-4 (IL-4) (20 ng/mL, Acro Biosystems), and 10% inactivated autologous plasma at a density of 1 × 10^6^ cells/mL. Half of the medium were changed every 48 hours with cytokine supplementation. FITC-conjugated ovalbumin (FITC-OVA, 50 μg/mL, Solarbio) was added on day 1, 3, 5, and 7 of DC culture, respectively, followed by 30-minute incubation. Cells were washed three times with cold PBS (300 × g, 5 min) to remove unbound FITC-OVA. Antigen uptake was quantified using a FACS Canto II flow cytometer (BD Biosciences).

### 2.3 DC vaccine preparation

Two MUC1 derived-antigenic peptides, Peptide 568 (GVTSAPDTRPAPGSTA) and Peptide 619 (TRPAPGSTAPPAHGVTSAPDTRPAPGSTAP), were synthesized by China Peptides Co., Ltd. (Shanghai, China). DC precursors (3 × 10^6^ cells/mL) were seeded into three 25 cm² flasks. On day 5 of differentiation, Peptide 568, Peptide 619 (500 ng/mL) and the same volume of culture medium were added to the three 25 cm² flasks, respectively. After 24 hours, all cells were stimulated with LPS (1 μg/mL, Selleck Biosystems) and TNF-α (50 ng/mL, Acro Biosystems) to induce maturation. DCs harvested on day 7 served as antigen-loaded vaccines, while antigen-unloaded mDCs were used as controls.

### 2.4 CCK8 assay for T lymphocyte proliferation

The stimulatory effect of DC vaccines on proliferation of naive T lymphocyte was assessed using the stimulation index (SI). Naive T lymphocytes were seeded in 96-well plates at a density of 2 × 10^5^ cells/well and co-cultured with antigen-loaded DCs at effector-to-target (E:T) ratios of 1:5, 1:10, and 1:20. Controls included antigen-unloaded mature DCs groups, T lymphocyte-only groups, and culture medium-only groups, with triplicate wells per condition and a total volume of 200 μL per well. After 48 hours of incubation at 37°C, 20 μL of CCK8 (Beijing Tongren Chemical Technology Co., Ltd.) was added to each well and incubated for 2 hours. Absorbance (A) was measured at 450 nm using a microplate reader. SI was calculated as: SI= (A _experimental group_ - A _DCs-only group_)/(A _lymphocyte-only group_ - A _culture medium-only group_) × 100%.

### 2.5 The induction of cytotoxic T lymphocyte

Naive T lymphocytes (1 × 10^7^) were co-cultured with DCs loaded with two distinct antigen peptides or antigen-unloaded mDCs at a T:DC ratio of 10:1. The cells were cultured with fresh X-VIVO 15 medium containing 5% heat-inactivated autologous plasma and 500 IU/mL IL-2. After 7 days of co-culture, suspended cells were harvested as CTLs activated by the respective DCs. T lymphocytes cultured without DCs for 7 days served as the PBS control group.

### 2.6 Flow cytometric analysis of surface markers

DCs collected on days 0, 5, and 7 were stained with anti-human CD11c-FITC, CD14-FITC, HLA-DR-PerCP, CD83-PE, CD80-PE, and CD86-APC antibodies. Isotype controls included mouse IgG1-PE, IgG1-APC, IgG1-PerCP, and IgG1-FITC. CTLs were stained with anti-human CD3-FITC, CD4-APC, and CD8-PE. All samples were incubated at 4°C in the dark for 15 minutes, washed with PBS, and analyzed using a FACS Canto II flow cytometer.

### 2.7 ELISA and lymphocyte cytotoxicity assay

Supernatants from DC vaccines and corresponding CTLs cultured for 7 days were collected. The secretion levels of IL-12p70 by DC vaccines and IFN-γ by CTLs were measured using ELISA kits (R&D Systems) according to the manufacturers’ instructions. CTLs activated by DC vaccines were used as effector cells, while PANC-1, BXPC-3, and MIA PaCa-2 cells served as target cells. Target cells (1 × 10^4^) were co-cultured with effector cells at effector-to-target ratios of 5:1, 10:1, 20:1, and 40:1. Control groups included tumor cell spontaneous release group, effector cell spontaneous release group, and tumor cell maximum lysis group. After 12 hours of co-culture, cytotoxicity was assessed using the lactic dehydrogenase (LDH) release assay (Dojindo, Kumamoto, Japan). Cytotoxicity (%) was calculated as follows: Cytotoxicity (%) = (A value of effector-to-target group - A value of tumor cell spontaneous release group - A value of effector cell spontaneous release group)/(A value of tumor cell maximum lysis group - A value of tumor cell spontaneous release group) × 100%.

### 2.8 Bioinformatics analysis of MUC1 expression and survival in pancreatic cancer patients

mRNA-seq data along with clinical data from 178 pancreatic cancer patients were downloaded from the TCGA database. mRNA-seq data from 171 normal individuals were obtained from the GTEx database. RNA-seq probe data from TCGA and GTEx were annotated and converted into gene expression matrices using R Studio. The ggplot2 package was used to analyze and visualize MUC1 gene expression in pancreatic cancer and normal tissues. Patients were divided into high and low MUC1 expression groups based on the median expression level. Kaplan-Meier survival analysis was performed, and survival curves were plotted using the ggsurvplot package.

### 2.9 Western blot analysis of MUC1 expression in pancreatic cancer cell lines

PANC-1, BXPC-3, and MIA PaCa-2 cells were lysed using RIPA Lysis buffer, and the lysates were centrifuged at 12,000 × g for 15 minutes at 4°C. Protein concentration was determined using a BCA protein assay kit (Beyotime). Protein samples were mixed with loading buffer, boiled for 10 minutes, and separated using 12.5% SDS-polyacrylamide gels. Proteins were electro-transferred onto a polyvinylidene fluoride membrane (Beyotime). The membranes were then blocked with QuickBlock™ blocking buffer for 15 minutes at room temperature (Beyotime). Subsequently, the membranes were incubated overnight at 4°C with different primary antibodies: rabbit anti-human MUC1 (1:1000, Beyotime) and rabbit anti-human β-Actin (1:1000, Beyotime). After washing with TBST, the membranes were incubated with horseradish peroxidase-conjugated secondary antibodies: goat anti-rabbit IgG (1:1000, Beyotime) for 1 hour at room temperature. The protein signal was visualized using the Chemiluminescence kit (Beyotime) and Bio-Rad ChemiDoc imaging system. After the western blotting procedure, ImageJ software was used to quantify the gray value of western blot bands.

### 2.10 Evaluation of immune reconstitution in huHSC-M-NSG mice

On day 28 after hematopoietic stem cell (HSC) transplantation, immune reconstitution was assessed by collecting 100 µL of peripheral blood from four randomly selected huHSC-M-NSG mice. Red blood cells were lysed in the dark for 15 minutes, and the remaining cells were washed once with PBS and resuspended in 300 µL PBS. The cells were stained with anti-human CD45-APC, anti-mouse CD45-FITC, and the corresponding isotype control antibodies. After incubation at 4°C in the dark for 15 minutes, the cells were washed and resuspended in 150 µL PBS for flow cytometry analysis. The percentage of human CD45^+^ cells (hCD45%) was calculated as hCD45% = hCD45/(hCD45 + mCD45) × 100%. Immune reconstitution was considered successful when hCD45% ≥ 25% (15).

### 2.11 Therapeutic vaccination of DC vaccines in huHSC-M-NSG mice

The study used human pancreatic cancer PANC-1 xenograft models in 16–18 weeks old female huHSC-M-NSG mice. PANC-1 tumor tissue was cut into 2.0 mm × 2.0 mm × 2.0 mm pieces and subcutaneously implanted into the left flank of the mice (Day 0). Then, the mice were randomly divided into three groups with six mice per group: PBS control group, antigen-unloaded DC group, and DC vaccine group loaded with Peptide 619. On day 4, the first vaccination was administered intradermally at a dose of 1 × 10^6^ cells/50 µL, with the control group receiving an equal volume of PBS or antigen-unloaded DC. The second vaccination was given on day 7 at a different site. Tumor volume was measured twice weekly using calipers, and calculated as 0.5 × length × width². Tumor growth inhibition rate (%) was calculated as (1 - tumor volume of treatment group/tumor volume of control group) × 100%. When the tumor volume reached 1000 mm³, the mice were euthanized after exposure to pentobarbital overdose (50 mg/kg), and the tumors were excised, weighed, and photographed. Tumor weight inhibition rate (%) was calculated as (1 - tumor weight of treatment group/tumor weight of control group) × 100%. Peripheral blood and tumor tissue were collected for analysis the ratios of CD4^+^ and CD8^+^ T cell and the infiltration of hCD45^+^ cell by flow cytometry, respectively.

### 2.12 Statistical analysis

**Figure 1:**
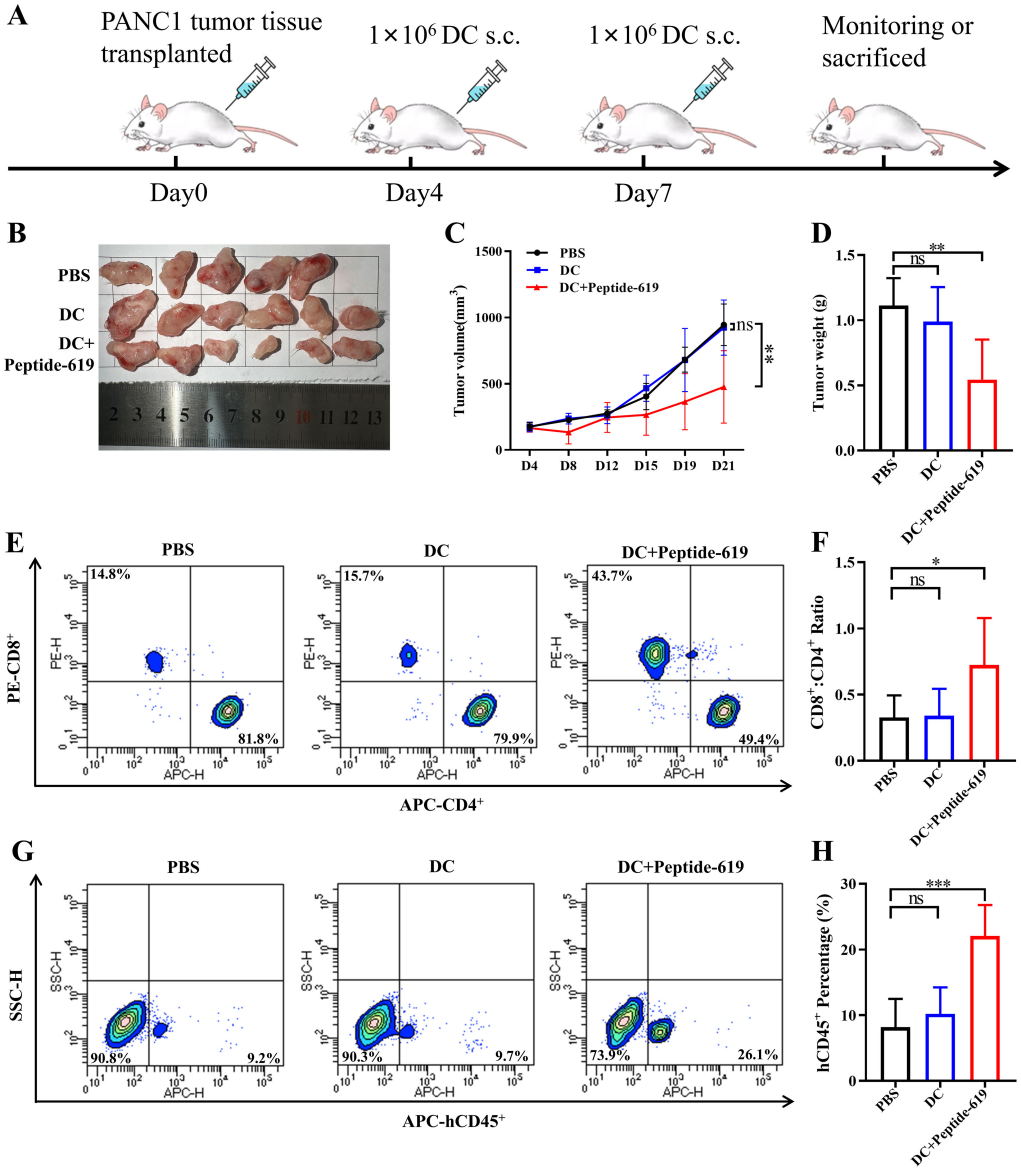
*In vivo* therapeutic effect mediated by antigen peptide 619 loaded DCs vaccine. **(A)** Tumor inoculation and DC vaccination schedule. s.c., subcutaneous injection. **(B)** Tumor size in each group (n=6). **(C)** Tumor volume growth curves in each group (n=6). **(D)** Tumor weight on day 21 in each group (n=6). **(E)** Percentage of CD4^+^ T cells and CD8^+^ T cells in peripheral blood. **(F)** Ratio of CD8^+^ T cells/CD4^+^ T cells in peripheral blood (n=6). **(G, H)** Percentage of hCD45^+^ cells infiltration in tumor tissues (n=6). All data are expressed as mean ± SD, *P < 0.05, **P < 0.01, ***P < 0.001; ns, not significant.

Statistical analysis was performed using GraphPad Prism 9.5. Continuous data are presented as mean ± SD. Normality was assessed using the Shapiro-Wilk test. For comparisons between two groups, unpaired Student’s t-tests were used for normally distributed data; otherwise, the Mann-Whitney U test was applied. Multiple group comparisons were conducted using one-way ANOVA (with Tukey’s *post hoc* test) for normally distributed data, or the Kruskal-Wallis test (with Dunn’s *post hoc* correction) for non-normally distributed data. In the animal study (**Figure 1**), each group initially had n = 6 mice. All analyses followed the intention-to-treat principle, including all originally enrolled animals. Missing longitudinal data due to animal death were handled by multiple imputation to minimize bias and loss of information. Survival differences between MUC1 high- and low-expression groups were compared using Cox proportional hazards regression. A two-sided *P* < 0.05 was considered statistically significant.

## 3 Results

### 3.1 Surface marker expression and antigen phagocytic uptake capacity of DCs

**Figure 2:**
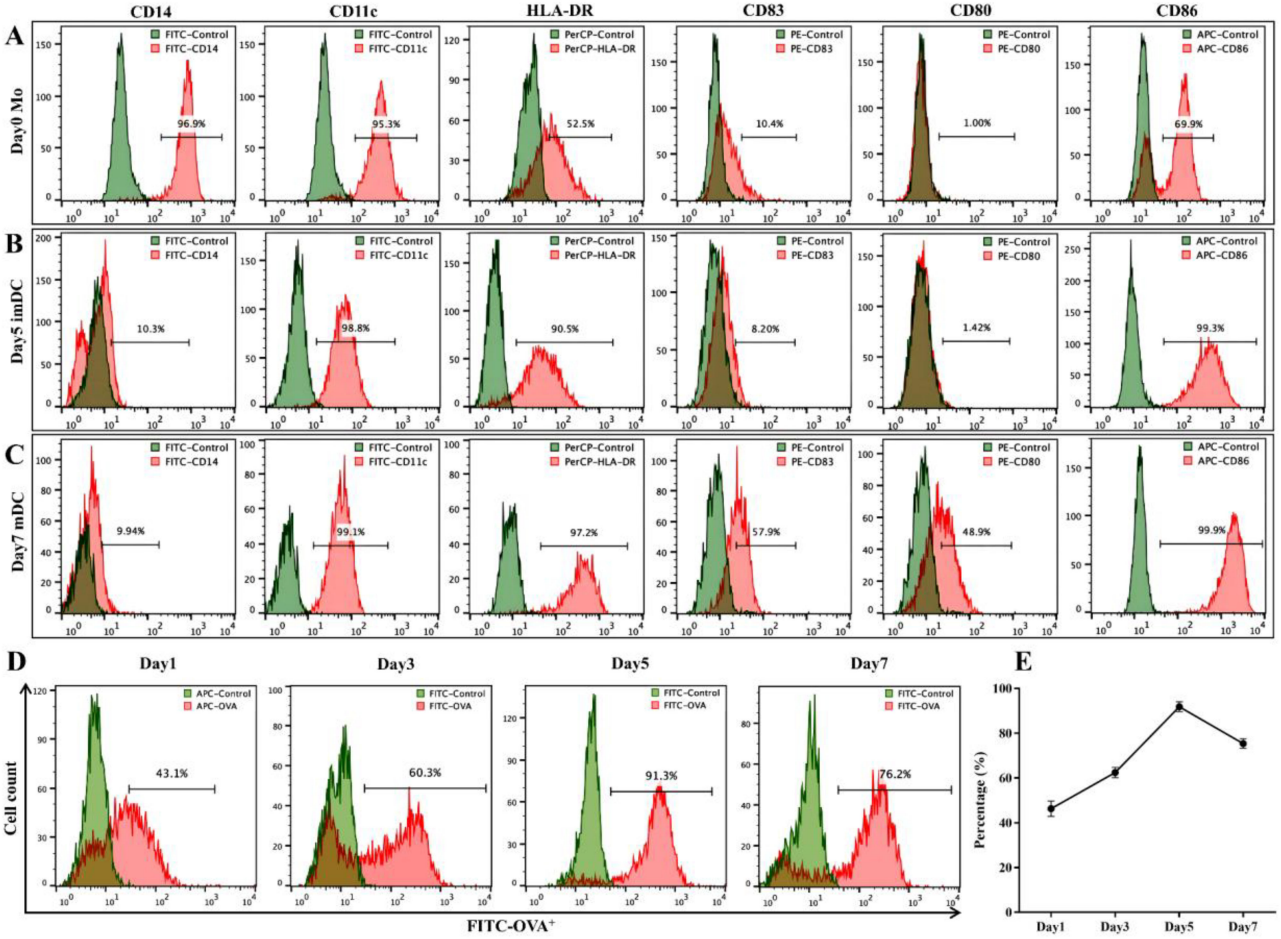
Surface marker expression and antigen uptake capacity of DCs. **(A)** The expression of CD14, CD11c, CD80, CD83, CD86 and HLA-DR on the surface of Mo; **(B)** The expression of CD14, CD11c, CD80, CD83, CD86 and HLA-DR on the surface of imDC; **(C)** The expression of CD14, CD11c, CD80, CD83, CD86 and HLA-DR on the surface of mDC; **(D, E)** DCs uptake of FITC-OVA was quantitatively measured by flow cytometry at different time points.

Flow cytometry was used to analyze the surface marker expression of monocytes (Mo), imDCs, and mDCs. The results showed that monocytes expressed high levels of CD14 and CD11c but low levels of CD83 and CD80 (**Figure 2A**). imDCs highly expressed CD11c, HLA-DR, and CD86 but had low expression of CD14, CD83, and CD80 (**Figure 2B**). In contrast, mDCs highly expressed CD11c, HLA-DR, CD83, CD80, and CD86 but had low expression of CD14 (**Figure 2C**). Additionally, DCs exhibited the strongest uptake capacity for FITC-OVA on day 5 of induction, reaching 91.80 ± 2.2% (**Figures 2D, E**).

### 3.2 Promotion of T cell proliferation and cytokine secretion by MUC1 antigen peptide-pulsed DCs

**Figure 3:**
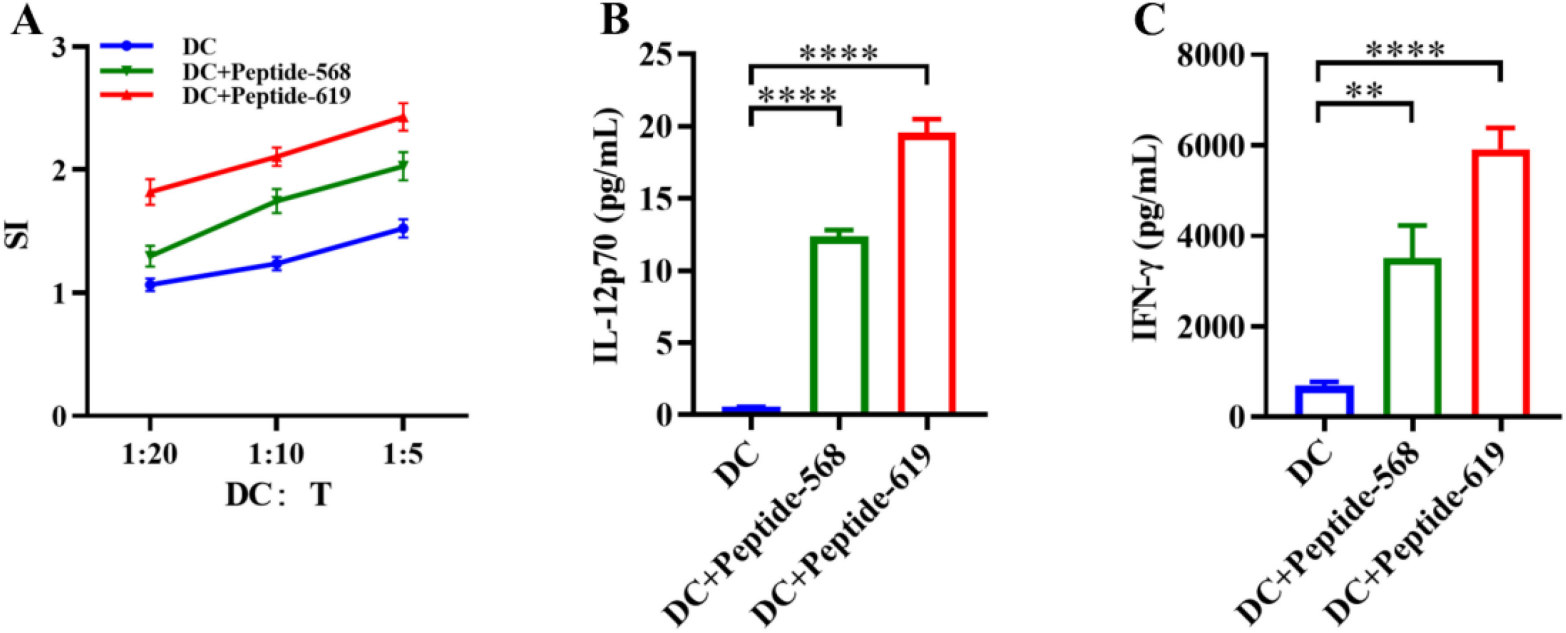
Enhancing effect of DC vaccines on T cell proliferation and cytokine secretion. **(A)** Lymphocyte proliferation activity promoted by DC vaccines. **(B)** IL-12p70 secretion by DCs. **(C)** IFN-γ secretion by CTLs. Data are representative of three independent experiments (n = 3 biological replicates; mean ± SD; **p < 0.01, ****p < 0.0001).

To evaluate the ability of MUC1 antigen peptide-pulsed DCs to promote T cell proliferation, a T lymphocyte stimulation assay was performed. The results demonstrated that DC vaccines loaded with MUC1 antigen peptides 568 and 619 significantly enhanced lymphocyte proliferation (**Figure 3A**). Furthermore, ELISA was used to measure the secretion levels of IL-12p70 in the supernatant of MUC1 antigen peptide-loaded DCs and IFN-γ in CTLs. The results showed that DC vaccines loaded with MUC1 antigen peptides 568 and 619 induced significantly higher IL-12p70 secretion compared to the antigen-unloaded DC control group (**Figure 3B**). Additionally, CTLs activated by DC vaccines exhibited stronger IFN-γ secretion (**Figure 3C**), further validating their ability to enhance CTL functional activity. The results shown are from three independent experiments (n=3 biological replicates) performed with cells from separate cultures.

### 3.3 The proportion of CD4^+^ and CD8^+^ cells in CTLs

**Figure 4:**
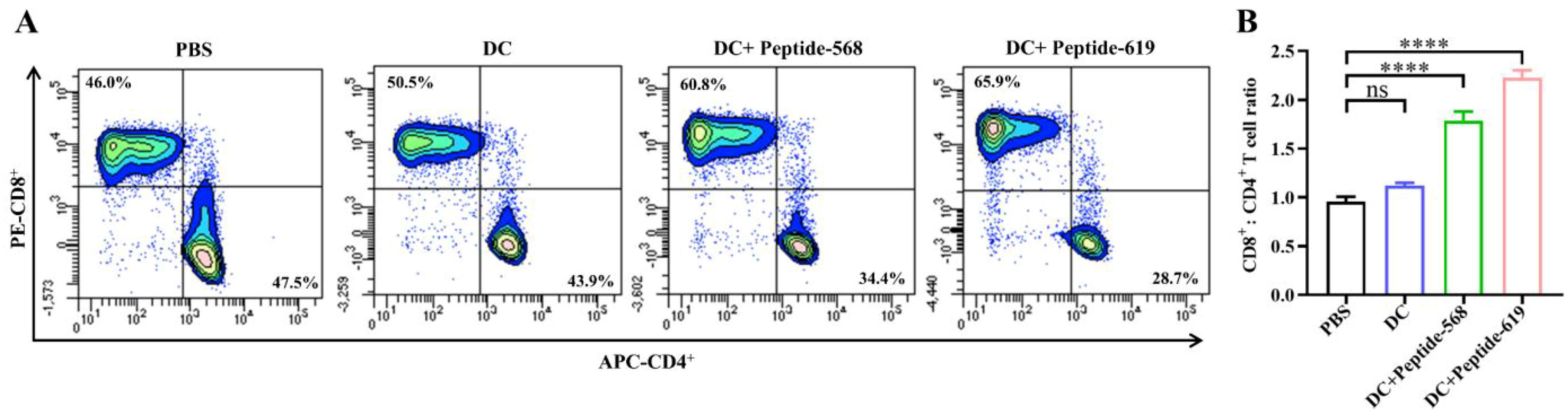
Proportion of CD8^+^ and CD4^+^ T cells in CTL populations. **(A, B)** Percentage of CD4^+^ and CD3^+^ T cells. **(C, D)** Percentage of CD8^+^ and CD3^+^ T cells. Data are representative of three independent experiments (n = 3 biological replicates; mean ± SD; ****p < 0.0001; ns, not significant).

To accurately evaluate the proportion of CD8^+^ and CD4^+^ T cells in CTLs activated by the DC vaccine, we co-cultured DC vaccines with autologous naïve T cells for 7 days and analyzed the percentages of CD4^+^ and CD8^+^ cells within the CD3^+^ T cell population using flow cytometry. The results demonstrated that, compared to the PBS control group, DC vaccines loaded with MUC1 antigenic peptides 568 and 619 significantly increased the proportion of CD8^+^ T cells and concurrently decreased that of CD4^+^ T cells (**Figures 4A, B**). The results shown are from three independent experiments (n=3 biological replicates) performed with cells from separate cultures. Detailed gating strategy and representative dot plots for flow cytometry can be found in the **Supplementary Material (Supplementary Figure S1)**.

### 3.4 *In vitro* cytotoxic activity of DC vaccine-activated CTLs against pancreatic cancer cells

**Figure 5:**
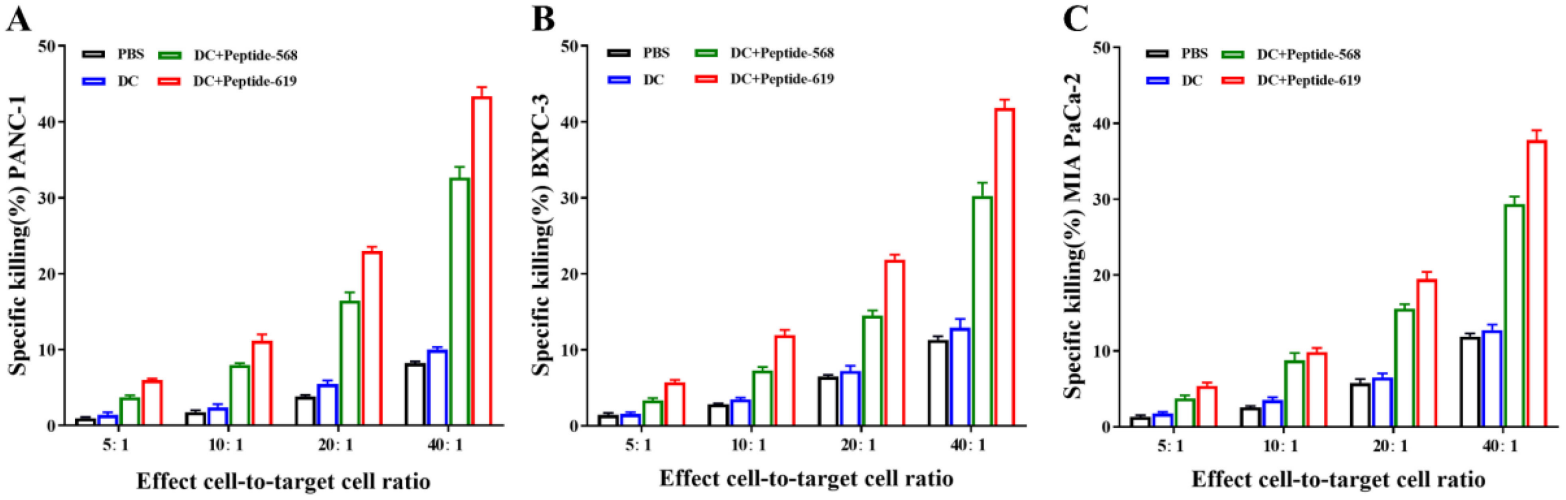
Anti-pancreatic cancer cells activity of DC vaccine-activated CTLs *in vitro*. **(A)** PANC-1. **(B)** BxPC-3. **(C)** MIA PaCa-2. Data are representative of three independent experiments (n = 3 biological replicates; mean ± SD).

The cytotoxic activity of CTLs induced respectively by PBS, antigen-unloaded DC, and MUC1 antigen peptides 568 and 619 loaded DC were evaluated against pancreatic cancer cell lines PANC-1, BXPC-3, and MIA PaCa-2. The results demonstrated that CTLs activated by DC vaccines loaded with MUC1 antigen peptides exhibited potent cytotoxic activity against all three pancreatic cancer cell lines (**Figures 5A–C**), with increasing cytotoxicity in a dose-dependent manner. At an effector-to-target (E:T) ratio of 40:1, the specific cytotoxic activities of CTLs activated by antigen peptide 568 loaded DC vaccines were 32.7 ± 1.1%, 30.3 ± 1.7%, and 29.3 ± 0.8% against PANC-1, BXPC-3, and MIA PaCa-2, respectively. Meanwhile, CTLs activated by antigen peptide 619 loaded DC vaccines exhibited cytotoxic activities of 43.4 ± 1.2%, 41.8 ± 1.1%, and 37.8 ± 1.3% against the same cell lines, respectively. The results shown are from three independent experiments (n=3 biological replicates) performed with cells from separate cultures.

### 3.5 Expression of MUC1 in pancreatic cancer and its correlation with survival

**Figure 6:**
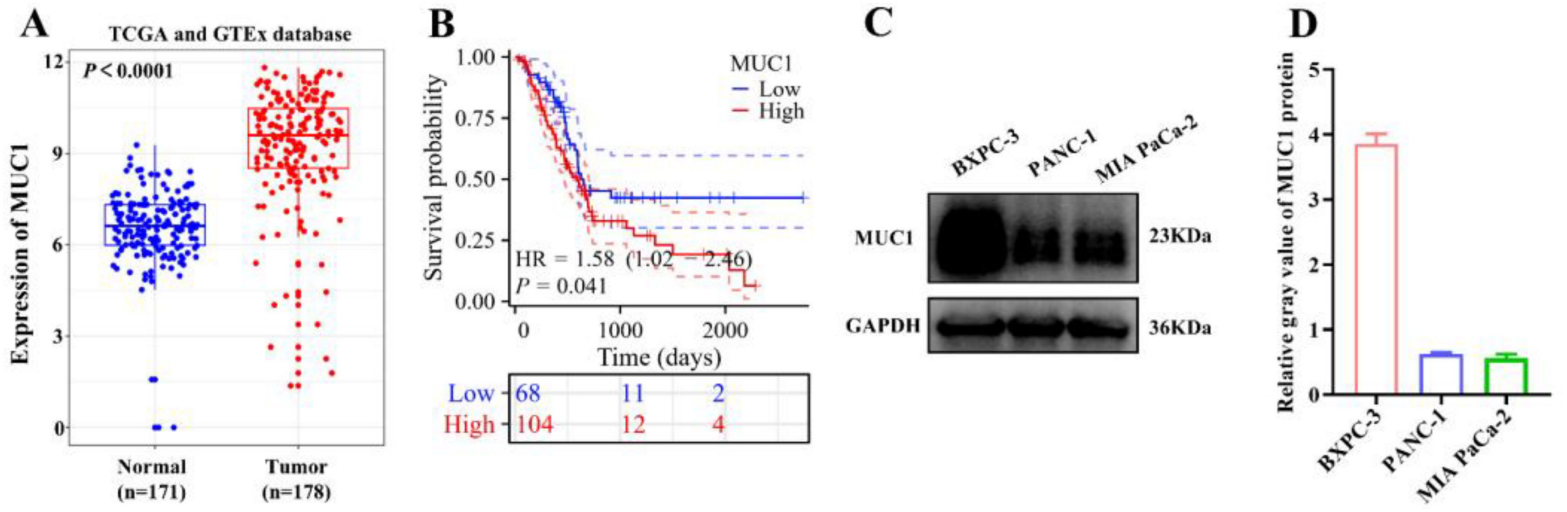
Expression of MUC1 in pancreatic cancer and its association with survival. **(A)** Differential expression of MUC1 in pancreatic cancer patients. **(B)** The correlation analysis between MUC1 expression and survival in pancreatic cancer patients. **(C)** Western blotting was performed to measure the expression of MUC1 in pancreatic cancer cell lines. **(D)** The statistical result of relative gray values.

Bioinformatics analysis was performed to evaluate the differential expression of MUC1 in pancreatic cancer patients compared to normal tissues. The results (**Figure 6A**) showed that MUC1 was significantly overexpressed in pancreatic cancer tissues (*P* < 0.0001). Further analysis revealed that high expression of MUC1 was associated with significantly reduction of overall survival in pancreatic cancer patients (*P* < 0.05, **Figure 6B**). Additionally, Western blot analysis confirmed the expression of MUC1 in PANC-1, BXPC-3, and MIA PaCa-2 cell lines (**Figures 6C, D**).

### 3.6 Evaluation of immune reconstitution in humanized mice

**Figure 7:**
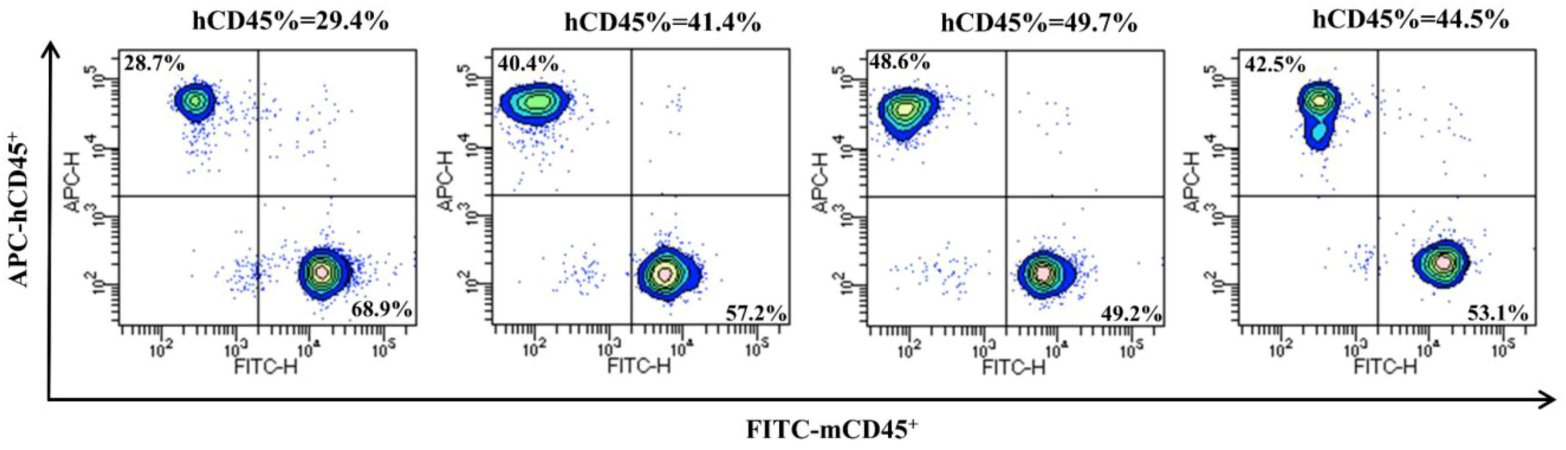
Evaluation of immune reconstitution in humanized mice.

Four huHSC-M-NSG mice were randomly selected, and the proportions of hCD45^+^ and mCD45^+^ cells in their peripheral blood were analyzed by flow cytometry. Immune reconstitution was considered successful when hCD45% ≥ 25%. The results showed that all mice met the immune reconstitution criteria (**Figure 7**). Detailed gating strategy and representative dot plots for flow cytometry can be found in the **Supplementary Material (Supplementary Figure S1)**.

### 3.7 The therapeutic effect of MUC1 antigen peptide 619-loaded DC vaccines on pancreatic cancer

On day 0, PANC-1 tumor tissue (2.0 mm × 2.0 mm × 2.0 mm) were subcutaneously implanted into the left flank of the huHSC-M-NSG mice, and DC vaccines were administered on day 4 (**Figure 1A**). During the experiment, one animal in the PBS control group died without apparent cause. On day 21 after tumor transplantation, all animals were euthanized, and tumors were excised, weighed, and photographed (**Figure 1B**). Compared to the PBS control group, the antigen peptide 619 loaded DC vaccine significantly inhibited tumor growth (*P* < 0.01, **Figures 1C, D**). Based on tumor volume measurement, the tumor growth inhibition rates on day 21 were 2.30% in the antigen-unloaded DC vaccine group and 49.6% in the antigen peptide 619-loaded DC vaccine group (**Figure 1C**). When calculated based on tumor weight, the inhibition rates were 11.15% and 51.4% for the antigen-unloaded and antigen peptide 619-loaded groups, respectively (**Figure 1D**). Notably, three mice in the antigen peptide 619-loaded group exhibited particularly high tumor weight inhibition rates of 73.0%, 77.50%, and 73.90%. Compared to the PBS control and antigen-unloaded DC groups, the antigen peptide 619 loaded DC vaccine group exhibited a significant increase in CD8^+^ T lymphocytes in peripheral blood (**Figures 1E, F**) and enhanced infiltration of hCD45^+^ cells in tumor tissues (**Figures 1G, H**). Detailed gating strategy and representative dot plots for flow cytometry can be found in the **Supplementary Material (Supplementary Figure S1)**.

## 4 Discussion

DCs as pivotal immune cells, are primarily responsible for antigen capture and presentation to activate naive T lymphocytes (16). imDCs specialize in antigen uptake and processing but exhibit limited T-cell activation capacity, functioning in a “surveillance” state, whereas mDCs focus on antigen presentation and naive T-cell priming (17). The optimal window for antigen loading onto DCs occurs between days 3 and 7 of culture (18). Our findings revealed that DCs cultured in GM-CSF and IL-4 exhibited the strongest antigen uptake capacity (91.80 ± 2.2%) on day 5, suggesting this timepoint as the optimal antigen-loading phase. mDCs specifically express surface markers such as HLA-DR, CD11c, CD83, CD86, and CD80 (19). For instance, the B7 family molecules CD80 and CD86 on DCs bind to the CD28 receptor on T cells, and their expression levels reflect DC maturation (20). In this study, the expression level of surface markers (CD80, CD86, CD83, HLA-DR, and CD11c) on mDCs progressively increased, while the marker of CD14 on monocytes decreased from 96.9% (day 0) to 9.94% (day 7). These phenotypic results demonstrate that antigen-loaded, maturation factor-treated DCs possess robust antigen-presenting capacity, co-stimulatory potential, and the ability to activate antitumor immunity.

MUC1 peptide-loaded DCs displayed elevated secretion levels of IL-12p70 and significantly enhanced T-cell proliferation and IFN-γ secretion. IL-12p70 secreted by DCs promotes Th1-type immune responses by activating T cells and natural killer (NK) cells, thereby amplifying antitumor immunity (21–23). Concurrently, IFN-γ secreted by T cells augments the cytotoxic activity of CD8^+^ T cells and NK cells, enhances macrophage-mediated tumor suppression, and orchestrates immune cell crosstalk, collectively inhibiting tumor growth and metastasis. CD8^+^ T cells, commonly referred to as CTLs, are primarily responsible for directly recognizing and eliminating infected or malignant cells (24). They function through recognizing antigenic peptides presented by MHC class I molecules on target cell surfaces and releasing cytotoxic molecules such as perforin and granzymes to induce apoptosis (25). In contrast, excessive CD4^+^ regulatory T cells (Tregs) may disrupt immune balance by secreting inhibitory cytokines (e.g., IL-10, IL-35, TGF-β), thereby suppressing CTL and NK cell activity (26). In this study, MUC1 peptide-loaded DC vaccines significantly increased the CD8^+^/CD4^+^ T-cell ratio, further validating their potential to enhance antitumor immunity.

Bioinformatics analysis revealed that MUC1 was markedly overexpressed in pancreatic cancer tissues, and this overexpression correlated with reduced overall survival in patients, aligning with prior reports implicating MUC1 in tumor immune evasion and microenvironmental regulation (27, 28). Western blot confirmed stable MUC1 expression in multiple pancreatic cancer cell lines (PANC-1, BxPC-3, MIA PaCa-2), providing a molecular rationale for MUC1-targeted immunotherapy. Notably, DCs loaded with Peptide 619 (a longer sequence containing multiple repetitive epitopes) exhibited superior efficacy in promoting T-cell proliferation and CTL-mediated cytotoxicity compared to Peptide 568, likely due to enhanced DC processing and broader T-cell receptor (TCR) repertoire activation.

In the huHSC-M-NSSG humanized mouse model, mice vaccinated with the antigen peptide 619-loaded DC vaccine exhibited tumor volume and weight inhibition rates of 49.6% and 51.4%, respectively. Notably, three mice in this group showed particularly high tumor weight inhibition rates of 73.0%, 77.50%, and 73.90%, demonstrating markedly superior efficacy compared to the blank DC control group. This study is the first to demonstrate the therapeutic efficacy of MUC1-targeted DC vaccines in a humanized immune model. In theory, increased peripheral CD8^+^ T-cell proportions and enhanced hCD45^+^ cell infiltration in tumors suggested vaccine-driven recruitment of effector T cells into the tumor microenvironment.

However, this study has several limitations. (1) Although HLA-A*02:01/MUC1 tetramer staining is the gold standard for identifying antigen-specific CD8^+^ T cells, this analysis was not performed due to constraints in the experimental timeline and reagent availability. (2) In the initial phase of this study, cytokine detection focused primarily on key Th1-type immune response factors such as IFN-γ and the dendritic cell maturation-related factor IL-12p70, without systematically evaluating the expression levels of regulatory cytokines such as IL-10 and TGF-β. This limits a comprehensive understanding of the nature of the vaccine-induced immune response. (3) The clonal composition of the TCR repertoire induced by peptide 619 and the immunogenic characteristics of its dominant epitopes remain unclarified. To address these limitations, we plan to prioritize tetramer staining experiments in follow-up studies to provide direct evidence of antigen-specific T-cell responses. Concurrently, we will employ multiplex cytokine assays to comprehensively analyze cytokine secretion profiles and further explore combination strategies of this DC vaccine with immune checkpoint inhibitors, such as anti-PD-1 antibodies.

In conclusion, this study highlights the therapeutic potential of MUC1 peptide-loaded DC vaccines in pancreatic cancer. By eliciting tumor-specific immunity, DC vaccines effectively suppress tumor growth and remodel the immunosuppressive microenvironment. Future efforts should optimize vaccine design and explore synergistic regimens with other immunotherapies to improve clinical outcomes for pancreatic cancer patients.

## Data Availability

Publicly available datasets were analyzed in this study. This data can be found here: https://portal.gdc.cancer.gov/.
