# Supplementary material for: MUC1 peptide-loaded dendritic cell vaccine boosts antitumor immunity in pancreatic cancer
Source: Front Immunol. 2026 Jan 13;16:1752861. doi: 10.3389/fimmu.2025.1752861 (PMC12834808; doi:10.3389/fimmu.2025.1752861)
Supplement: Supplementary file 1 [file SupplementaryFile1.docx]

Supplementary Material

# Supplementary Figures


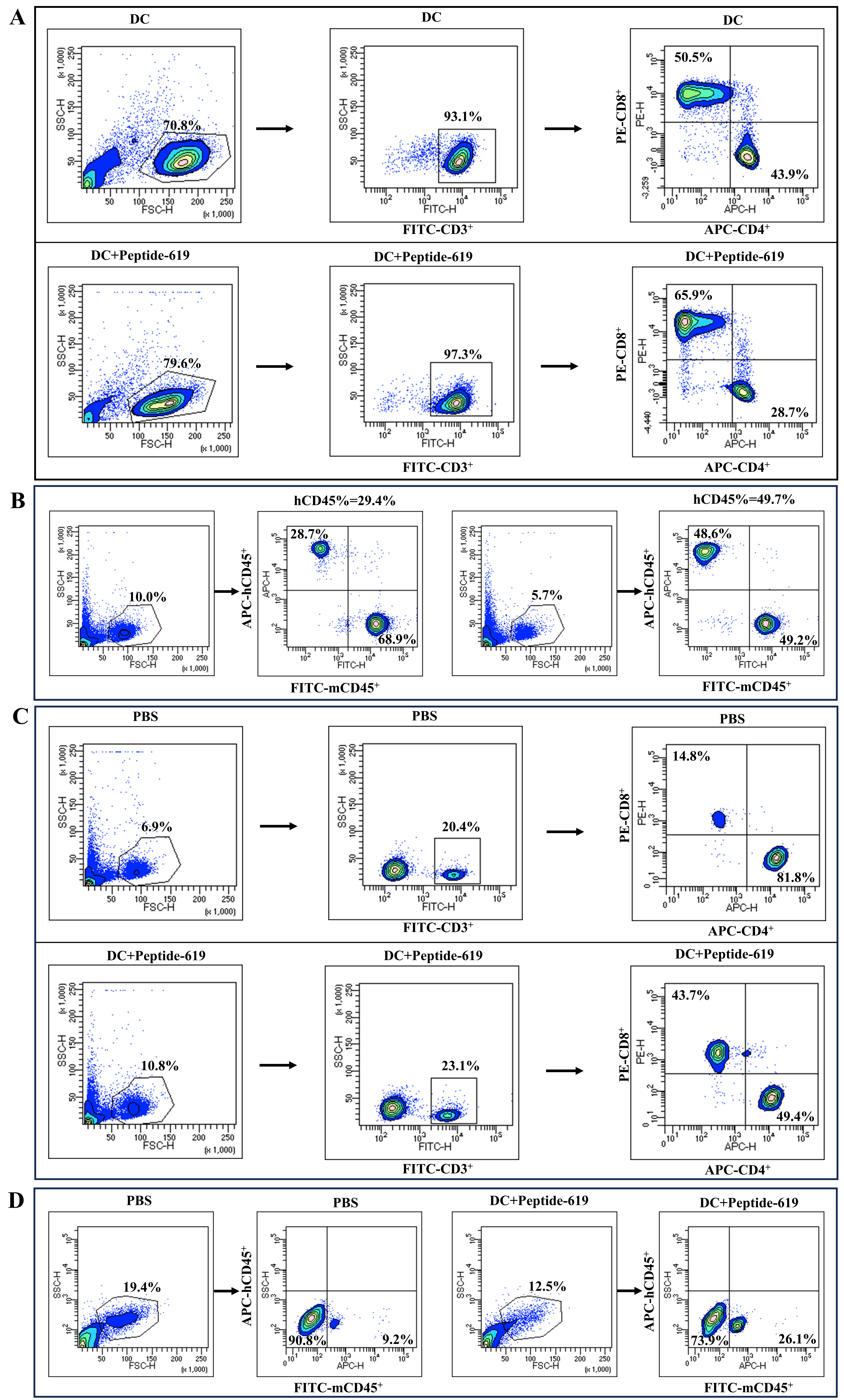


**Supplementary Figure S1.** Gating strategy and representative scatter plots in flow cytometry analysis. (A) Gating strategy and representative plots showing the proportion of CD4⁺ and CD8⁺ cells in CTLs; (B) Gating strategy and representative plots showing the percentage of hCD45⁺ and mCD45⁺ cells in peripheral blood after immune reconstitution; (C) Gating strategy and representative plots for the ratio of CD8⁺ to CD4⁺ T lymphocytes in peripheral blood from the PBS control and DC vaccine groups; (D) Gating strategy and representative plots for the proportion of infiltrating hCD45⁺ cells in tumor tissue from mice in the PBS control and DC vaccine groups.
